# Benz­yl(meth­yl)phosphinic acid

**DOI:** 10.1107/S1600536810024116

**Published:** 2010-06-26

**Authors:** Cécile Fougère, Erwann Guénin, Pascal Retailleau, Carole Barbey

**Affiliations:** a Université Paris-Nord, UFR-SMBH, Laboratoire de Chimie, Structures, Propriétés de Biomatériaux et d’Agents Thérapeutiques, (FRE 3043 CNRS), 74 rue M. Cachin, 93017 Bobigny Cedex, France; b Service de Cristallochimie, Institut de Chimie des Substances Naturelles, CNRS, 1 Av. de la Terrasse, 91198 Gif sur-Yvette cedex, France

## Abstract

The title compound, C_8_H_11_O_2_P, is a phosphinic compound with a tetra­coordinate penta­valent P atom. The phosphinic function plays a predominant role in the cohesion of the crystal structure, both by forming chains along the *b *axis *via* strong inter­molecular O—H⋯O hydrogen bonds and by cross-linking these chains perpendicularly *via* weak inter­molecular C—H⋯O hydrogen bonds, generating a two-dimensional network parallel to (001).

## Related literature

For general background to phosphinic compounds and their biological applications, see: Ye *et al.* (2007 ▶); Abrunhosa-Thomas *et al.* (2007 ▶); Wang *et al.* (2009 ▶). For their inhibitor properties and use as anti­bacterial agents, see: Boyd *et al.* (1994 ▶); Matziari *et al.* (2004 ▶); Ryglowski & Kafarski (1996 ▶). For the preparation of phosphinic acid, see: Montchamp (2005 ▶); Dingwall *et al.* (1989 ▶); Fougère *et al.* (2009 ▶). For related structures, see: Frantz *et al.* (2003 ▶); Langley *et al.* (1996 ▶); Cai *et al.* (2003 ▶); Meyer *et al.* (2003 ▶).
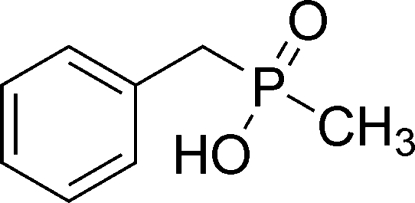


## Experimental

### 

#### Crystal data

C_8_H_11_O_2_P
*M* *_r_* = 170.14
Monoclinic, 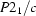

*a* = 9.3075 (4) Å
*b* = 8.2526 (4) Å
*c* = 11.8890 (4) Å
β = 108.657 (3)°
*V* = 865.22 (6) Å^3^
*Z* = 4
Mo *K*α radiation
μ = 0.27 mm^−1^
*T* = 293 K
0.60 × 0.25 × 0.06 mm

#### Data collection

Nonius KappaCCD diffractometer
10548 measured reflections
1767 independent reflections
1320 reflections with *I* > 2σ(*I*)
*R*_int_ = 0.050

#### Refinement

*R*[*F* ^2^ > 2σ(*F* ^2^)] = 0.038
*wR*(*F* ^2^) = 0.096
*S* = 1.05
1767 reflections
100 parameters
H-atom parameters constrained
Δρ_max_ = 0.18 e Å^−3^
Δρ_min_ = −0.29 e Å^−3^

### 

Data collection: *COLLECT* (Hooft, 1998 ▶); cell refinement: *HKL* (Otwinowski & Minor, 1997 ▶); data reduction: *COLLECT*; program(s) used to solve structure: *SHELXS97* (Sheldrick, 2008 ▶); program(s) used to refine structure: *SHELXL97* (Sheldrick, 2008 ▶); molecular graphics: *ORTEP-3 for Windows* (Farrugia, 1997 ▶) and *PLATON* (Spek, 2009 ▶); software used to prepare material for publication: *WinGX* (Farrugia, 1999 ▶) and *CrystalBuilder* (Welter, 2006 ▶).

## Supplementary Material

Crystal structure: contains datablocks global, I. DOI: 10.1107/S1600536810024116/dn2573sup1.cif

Structure factors: contains datablocks I. DOI: 10.1107/S1600536810024116/dn2573Isup2.hkl

Additional supplementary materials:  crystallographic information; 3D view; checkCIF report

## Figures and Tables

**Table 1:** Hydrogen-bond geometry (Å, °)

*D*—H⋯*A*	*D*—H	H⋯*A*	*D*⋯*A*	*D*—H⋯*A*
O1—H1⋯O2^i^	0.82	1.70	2.493 (2)	162
C7—H7⋯O2^ii^	0.93	2.54	3.377 (3)	151

Symmetry codes: (i) 
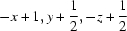
; (ii) 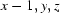.

**Fig. 1.**
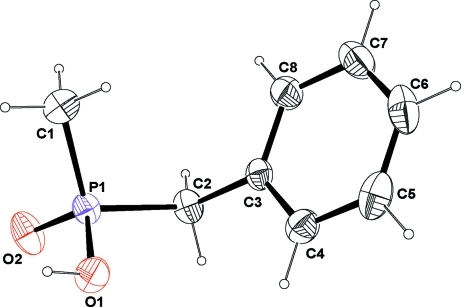
Molecular View of the title compound. Displacement ellipsoids are drawn at the 30% probability level.

**Fig. 2.**
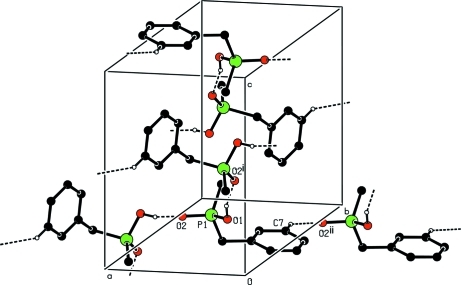
Molecular packing view with intermolecular hydrogen bonds drawn as dashed lines. H atoms not involved in hydrogen bondings have been omitted for clarity. [Symmetry codes: (i) -x+1, y+1/2, -z+1/2; (ii) x-1, y, z]

## supplementary crystallographic information

### Comment

The title compound, C_8_H_11_O_2_P, belongs to the phosphinic acid family (*R*'P(O)OHR''). These compounds are important substrates in the study of biochemical processes, and those comprising tetracoordinate pentavalent phosphorus are widely used as biologically active compounds. Mimics of amino acids in which the carboxylic function is replaced by phosphorus analogues have attracted particular interest. Among these phosphorus functions, phosphinic acid moiety is an excellent mimic of the tetrahedral transition state of amid bond hydrolysis and is more stable than phosphonic or phosphonamidic isosters. Thus, phosphinic compounds occupy an important place and reveal diverse and interesting biological and biochemical properties (Ye *et al.*, 2007; Abrunhosa-Thomas *et al.*, 2007; Wang *et al.*, 2009): phosphinic peptides have been reported to be potent inhibitors of several matrixins (MMPs) (Matziari *et al.*, 2004) and are widely studied as antibacterial agents, enzyme inhibitors, haptens for catalytic antibodies, or anti HIV agents (Boyd *et al.*, 1994; Ryglowski & Kafarski, 1996).

The development of methods for the preparation of phosphinic acids is so important and currently attracting growing interest (Montchamp, 2005; Dingwall *et al.*, 1989). The most commonly employed methods to prepare phosphinic acids suffer from several limitations: large excess of reagents, difficulties to avoid formation of symmetrically disubstituted phosphinic acids, handling difficulties of some starting materials. A new synthesis of unsymmetrical phosphinic acids *R*'P(O)OHR'' was performed. The first P—C bond formation was achieved using a base-promoted H-phosphinate alkylation from a protected H-phosphinate, easier and safer to handle. A one pot methodology was developed for the second P–C bond formation involving sila-Arbuzov reaction (Fougère *et al.*, 2009).

An *ORTEP* plot of the molecule is given in Fig. 1. Geometric parameters are in the usual ranges, *e.g.*; typical P = O, P—O and P—C bonds as it was found earlier in phosphonic acid crystal structures (Langley *et al.*, 1996; Frantz *et al.*, 2003; Meyer *et al.*, 2003; Cai *et al.*, 2003 ).

In the crystal packing, one molecule is linked to two adjacent symmetric molecules *via* strong intermolecular O–H···O==P hydrogen bonds (Table 1). These hydrogen bonds between phosphinic groups built an infinite intermolecular hydrogen-bond network along the *b* direction (Fig. 2), forming chains of molecules. These chains are perpendicularly cross-linked *via* weak hydrogen bonds between C-H from the aromatic ring and O from the phosphinic group (Table 1, Fig 2), that give rise to a bidimensionnal organization parallel to the (001) plane. The packing of the structure can also be described as a bidimensionnal organization piled up to the third direction with hydrophobic functions face to face.

### Experimental

To benzyl phosphinate (20 mmol) in acetonitrile (20 ml), bromotrimethylsilane (7 equiv) was added under argon bubbling. The triethylamine (2 equiv) was added, followed 5 minutes later by the bromide derivatives (1 equiv). The mixture was cooled to 0°C and absolute ethanol was added to quench the reaction. After 30 min., the solvent was removed and the residue was taken up in distilled water and extracted with ethyl acetate. The organic layer was dried under MgSO_4_; filtrated and evaporated under reduced pressure to give the crude product. This product was taken up in water (20 ml) and washed with ether (3 *x* 20 ml), followed by a reversed phase column chromatography (water/methanol 1:1) to give a white solid with high yield (76%). Single crystals suitable for X-ray structure analysis could be obtained by slow evaporation of a concentrated water/methanol (1/1) solution at room temperature.

### Refinement

All Hydrogen atoms attached to C atoms were fixed geometrically and treated as riding with C—H = 0.93 Å (aromatic), 0.96 Å (methylene) or 0.97 Å (secondary CH2 group) with *U*_iso_(H) = 1.2 *U*_eq_(C) (aromatic) or 1.5 *U*_eq_(C) for others. H atom of the hydroxyl was located in difference Fourier syntheses and was treated in the last stage of refinement as riding on it parent O atom with O—H = 0.82 Å and *U*_iso_(H) = 1.2 *U*_eq_(O).

### Crystal data

**Table:**

C_8_H_11_O_2_P	*F*(000) = 360
*M**_r_* = 170.14	*D*_x_ = 1.306 Mg m^−^^3^
Monoclinic, *P*2_1_/*c*	Mo *K*α radiation, λ = 0.71070 Å
Hall symbol: -P 2ybc	Cell parameters from 1896 reflections
*a* = 9.3075 (4) Å	θ = 0.4–26.4°
*b* = 8.2526 (4) Å	µ = 0.27 mm^−^^1^
*c* = 11.8890 (4) Å	*T* = 293 K
β = 108.657 (3)°	Parallelepipedic, colourless
*V* = 865.22 (6) Å^3^	0.60 × 0.25 × 0.06 mm
*Z* = 4	

### Data collection

**Table:**

Nonius KappaCCD diffractometer	1320 reflections with *I* > 2σ(*I*)
Radiation source: fine-focus sealed tube	*R*_int_ = 0.050
graphite	θ_max_ = 26.3°, θ_min_ = 2.3°
Detector resolution: 9 pixels mm^-1^	*h* = −11→11
φ and ω scans	*k* = −10→10
10548 measured reflections	*l* = −14→14
1767 independent reflections	

### Refinement

**Table:**

Refinement on *F*^2^	Primary atom site location: structure-invariant direct methods
Least-squares matrix: full	Secondary atom site location: difference Fourier map
*R*[*F*^2^ > 2σ(*F*^2^)] = 0.038	Hydrogen site location: inferred from neighbouring sites
*wR*(*F*^2^) = 0.096	H-atom parameters constrained
*S* = 1.05	*w* = 1/[σ^2^(*F*_o_^2^) + (0.0381*P*)^2^ + 0.2898*P*] where *P* = (*F*_o_^2^ + 2*F*_c_^2^)/3
1767 reflections	(Δ/σ)_max_ < 0.001
100 parameters	Δρ_max_ = 0.18 e Å^−^^3^
0 restraints	Δρ_min_ = −0.29 e Å^−^^3^

### Special details

**Table:**

Geometry. All e.s.d.'s (except the e.s.d. in the dihedral angle between two l.s. planes) are estimated using the full covariance matrix. The cell e.s.d.'s are taken into account individually in the estimation of e.s.d.'s in distances, angles and torsion angles; correlations between e.s.d.'s in cell parameters are only used when they are defined by crystal symmetry. An approximate (isotropic) treatment of cell e.s.d.'s is used for estimating e.s.d.'s involving l.s. planes.
Refinement. Refinement of *F*^2^ against ALL reflections. The weighted *R*-factor *wR* and goodness of fit *S* are based on *F*^2^, conventional *R*-factors *R* are based on *F*, with *F* set to zero for negative *F*^2^. The threshold expression of *F*^2^ > σ(*F*^2^) is used only for calculating *R*-factors(gt) *etc*. and is not relevant to the choice of reflections for refinement. *R*-factors based on *F*^2^ are statistically about twice as large as those based on *F*, and *R*- factors based on ALL data will be even larger.

### Fractional atomic coordinates and isotropic or equivalent isotropic displacement parameters (Å^2^)

**Table:**

	*x*	*y*	*z*	*U*_iso_*/*U*_eq_	
P1	0.37056 (5)	0.18896 (6)	0.22698 (4)	0.03697 (18)	
C1	0.3422 (3)	0.2500 (3)	0.36151 (18)	0.0574 (6)	
H11	0.3450	0.1566	0.4103	0.086*	
H12	0.2454	0.3023	0.3441	0.086*	
H13	0.4209	0.3241	0.4029	0.086*	
O1	0.36315 (16)	0.34293 (17)	0.15063 (12)	0.0487 (4)	
H1	0.4189	0.4128	0.1909	0.058*	
O2	0.51352 (16)	0.09571 (18)	0.24906 (16)	0.0606 (4)	
C2	0.2113 (2)	0.0707 (2)	0.14174 (19)	0.0440 (5)	
H21	0.2228	0.0488	0.0649	0.066*	
H22	0.2144	−0.0326	0.1814	0.066*	
C3	0.0567 (2)	0.1455 (2)	0.12169 (17)	0.0376 (5)	
C4	−0.0016 (3)	0.2590 (3)	0.03332 (17)	0.0485 (5)	
H4	0.0557	0.2914	−0.0140	0.058*	
C5	−0.1435 (3)	0.3248 (3)	0.0144 (2)	0.0618 (7)	
H5	−0.1812	0.4011	−0.0454	0.074*	
C6	−0.2295 (3)	0.2779 (3)	0.0838 (2)	0.0640 (7)	
H6	−0.3255	0.3218	0.0709	0.077*	
C7	−0.1734 (3)	0.1669 (3)	0.1716 (2)	0.0613 (7)	
H7	−0.2312	0.1352	0.2187	0.074*	
C8	−0.0310 (2)	0.1011 (3)	0.19124 (19)	0.0509 (6)	
H8	0.0064	0.0260	0.2519	0.061*	

### Atomic displacement parameters (Å^2^)

**Table:**

	*U*^11^	*U*^22^	*U*^33^	*U*^12^	*U*^13^	*U*^23^
P1	0.0327 (3)	0.0317 (3)	0.0455 (3)	0.0038 (2)	0.0113 (2)	0.0036 (2)
C1	0.0587 (14)	0.0668 (16)	0.0453 (12)	−0.0012 (12)	0.0147 (10)	0.0005 (12)
O1	0.0563 (9)	0.0378 (9)	0.0471 (8)	−0.0077 (7)	0.0097 (6)	0.0053 (6)
O2	0.0367 (8)	0.0452 (9)	0.0993 (12)	0.0114 (7)	0.0209 (8)	0.0051 (9)
C2	0.0418 (11)	0.0336 (11)	0.0557 (12)	−0.0018 (9)	0.0143 (9)	−0.0032 (9)
C3	0.0343 (10)	0.0351 (11)	0.0406 (10)	−0.0066 (8)	0.0081 (8)	−0.0062 (8)
C4	0.0508 (12)	0.0475 (13)	0.0442 (11)	−0.0013 (11)	0.0111 (10)	0.0027 (10)
C5	0.0581 (15)	0.0499 (15)	0.0602 (14)	0.0081 (12)	−0.0050 (11)	−0.0017 (12)
C6	0.0364 (12)	0.0625 (17)	0.0836 (17)	0.0008 (12)	0.0061 (12)	−0.0282 (15)
C7	0.0463 (13)	0.0689 (17)	0.0751 (16)	−0.0124 (13)	0.0284 (12)	−0.0164 (14)
C8	0.0471 (13)	0.0531 (14)	0.0525 (12)	−0.0080 (11)	0.0159 (10)	0.0034 (11)

### Geometric parameters (Å, °)

**Table:**

P1—O2	1.4859 (14)	C3—C4	1.382 (3)
P1—O1	1.5502 (14)	C3—C8	1.384 (3)
P1—C1	1.775 (2)	C4—C5	1.378 (3)
P1—C2	1.793 (2)	C4—H4	0.9300
C1—H11	0.9600	C5—C6	1.377 (4)
C1—H12	0.9600	C5—H5	0.9300
C1—H13	0.9600	C6—C7	1.361 (4)
O1—H1	0.8200	C6—H6	0.9300
C2—C3	1.514 (3)	C7—C8	1.381 (3)
C2—H21	0.9700	C7—H7	0.9300
C2—H22	0.9700	C8—H8	0.9300
			
O2—P1—O1	113.42 (9)	C4—C3—C8	118.07 (19)
O2—P1—C1	111.74 (11)	C4—C3—C2	121.23 (18)
O1—P1—C1	107.66 (10)	C8—C3—C2	120.70 (18)
O2—P1—C2	110.46 (9)	C5—C4—C3	120.9 (2)
O1—P1—C2	103.96 (9)	C5—C4—H4	119.5
C1—P1—C2	109.24 (10)	C3—C4—H4	119.5
P1—C1—H11	109.5	C6—C5—C4	120.1 (2)
P1—C1—H12	109.5	C6—C5—H5	119.9
H11—C1—H12	109.5	C4—C5—H5	119.9
P1—C1—H13	109.5	C7—C6—C5	119.6 (2)
H11—C1—H13	109.5	C7—C6—H6	120.2
H12—C1—H13	109.5	C5—C6—H6	120.2
P1—O1—H1	109.5	C6—C7—C8	120.5 (2)
C3—C2—P1	116.08 (14)	C6—C7—H7	119.8
C3—C2—H21	108.3	C8—C7—H7	119.8
P1—C2—H21	108.3	C7—C8—C3	120.8 (2)
C3—C2—H22	108.3	C7—C8—H8	119.6
P1—C2—H22	108.3	C3—C8—H8	119.6
H21—C2—H22	107.4		
			
O2—P1—C2—C3	174.91 (15)	C3—C4—C5—C6	0.0 (3)
O1—P1—C2—C3	−63.09 (17)	C4—C5—C6—C7	0.3 (4)
C1—P1—C2—C3	51.61 (18)	C5—C6—C7—C8	−0.1 (4)
P1—C2—C3—C4	81.4 (2)	C6—C7—C8—C3	−0.5 (4)
P1—C2—C3—C8	−99.0 (2)	C4—C3—C8—C7	0.8 (3)
C8—C3—C4—C5	−0.6 (3)	C2—C3—C8—C7	−178.8 (2)
C2—C3—C4—C5	179.01 (19)		

### Hydrogen-bond geometry (Å, °)

**Table:**

*D*—H···*A*	*D*—H	H···*A*	*D*···*A*	*D*—H···*A*
O1—H1···O2^i^	0.82	1.70	2.493 (2)	162
C7—H7···O2^ii^	0.93	2.54	3.377 (3)	151

Symmetry codes: (i) −*x*+1, *y*+1/2, −*z*+1/2; (ii) *x*−1, *y*, *z*.
